# Supplementary figures and images for: Field-based detection of bacteria using nanopore sequencing: Method evaluation for biothreat detection in complex samples
Source: PLoS One. 2023 Nov 28;18(11):e0295028. doi: 10.1371/journal.pone.0295028 (PMC10684088; doi:10.1371/journal.pone.0295028)

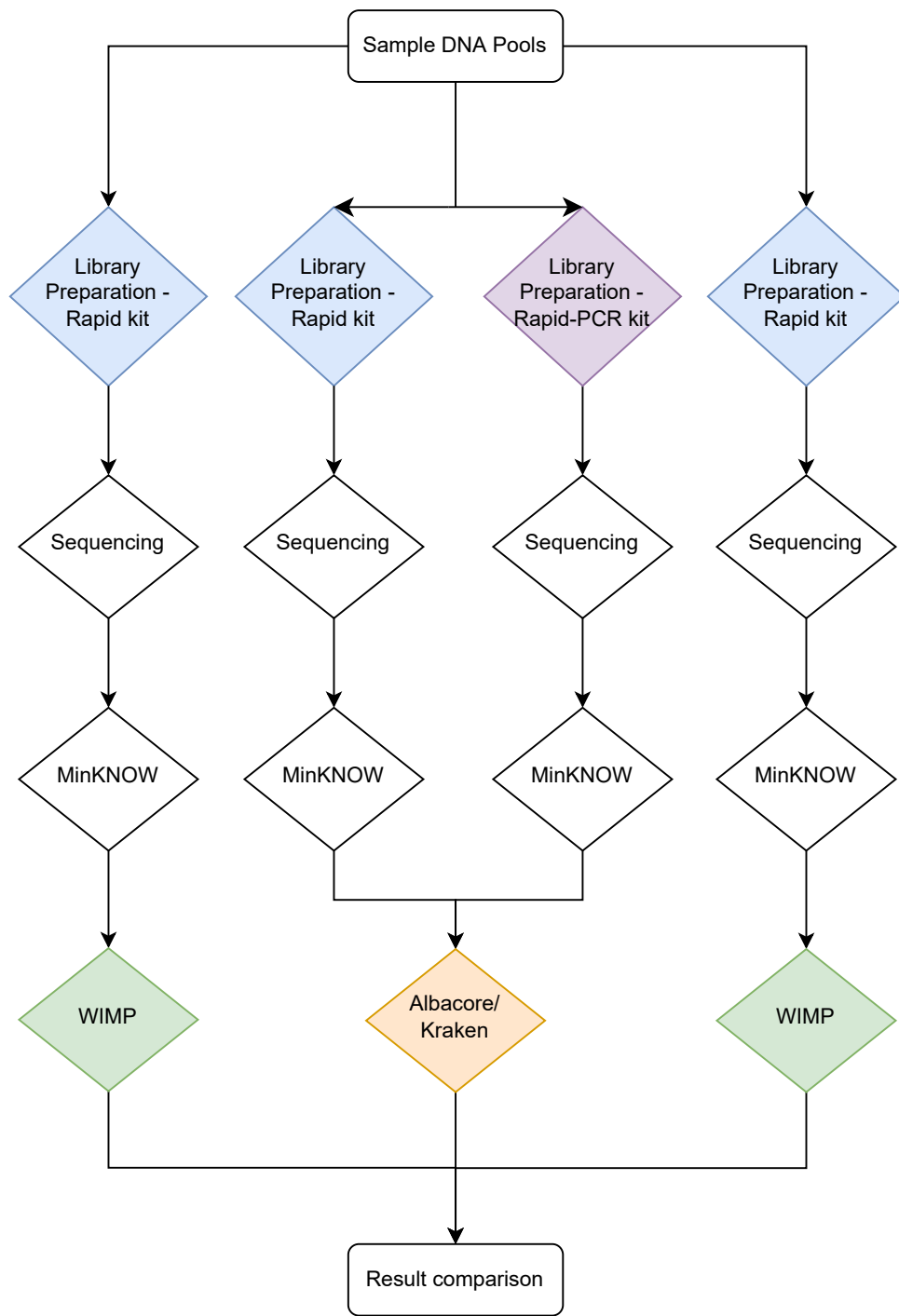

Supplement: S1 Fig — (PDF) [file pone.0295028.s001.pdf]
