# Supplementary material for: Field-based detection of bacteria using nanopore sequencing: Method evaluation for biothreat detection in complex samples
Source: PLoS One. 2023 Nov 28;18(11):e0295028. doi: 10.1371/journal.pone.0295028 (PMC10684088; doi:10.1371/journal.pone.0295028)
Supplement: Attachment — Submitted filename: revision-letter-20230714.docx [file pone.0295028.s002.docx]

Andrea Tyler

National Microbiology Laboratory

1015 Arlington St., Wpg, MB

R3E 36P

andrea.d.tyler@phac-aspc.gc.ca

August 1, 2023

Editorial Board

PLOS ONE

Submission: Field-based detection of bacteria using nanopore sequencing: method evaluation for biothreat detection in complex samples.

On behalf of the authors, I am pleased to submit the attached manuscript which has been modified as per the valuable suggestions provided by Reviewers. Included below, are specific comments related to the individual feedback for consideration.

On behalf of the authors involved in this work, we thank you again for your consideration and for providing feedback on this valuable work, and we look forward to our future correspondence.

Regards,

Andrea Tyler, PhD

Biologist – Public Health Agency of Canada

*RESPONSE TO REVIEWER COMMENTS*

1. Please ensure that your manuscript meets PLOS ONE's style requirements, including those for file naming. The PLOS ONE style templates can be found at

https://journals.plos.org/plosone/s/file?id=wjVg/PLOSOne_formatting_sample_main_body.pdf and

<https://journals.plos.org/plosone/s/file?id=ba62/PLOSOne_formatting_sample_title_authors_affiliations.pdf>

*We have updated the formatting to align with the provided guidelines. Note that these changes were not tracked in either the marked or unmarked drafts for ease of reading.*

2. Thank you for stating the following financial disclosure:

“This work was supported by the Canadian Safety and Security Program project, CSSP-2018-TI-2372”

Please state what role the funders took in the study.  If the funders had no role, please state: "The funders had no role in study design, data collection and analysis, decision to publish, or preparation of the manuscript."

*Thank you. We have added the above statement to the manuscript as suggested.*

Please include this amended Role of Funder statement in your cover letter; we will change the online submission form on your behalf.

3. In your Data Availability statement, you have not specified where the minimal data set underlying the results described in your manuscript can be found. PLOS defines a study's minimal data set as the underlying data used to reach the conclusions drawn in the manuscript and any additional data required to replicate the reported study findings in their entirety. All PLOS journals require that the minimal data set be made fully available. For more information about our data policy, please see http://journals.plos.org/plosone/s/data-availability.

Upon re-submitting your revised manuscript, please upload your study’s minimal underlying data set as either Supporting Information files or to a stable, public repository and include the relevant URLs, DOIs, or accession numbers within your revised cover letter. For a list of acceptable repositories, please see http://journals.plos.org/plosone/s/data-availability#loc-recommended-repositories. Any potentially identifying patient information must be fully anonymized.

Important: If there are ethical or legal restrictions to sharing your data publicly, please explain these restrictions in detail. Please see our guidelines for more information on what we consider unacceptable restrictions to publicly sharing data: http://journals.plos.org/plosone/s/data-availability#loc-unacceptable-data-access-restrictions. Note that it is not acceptable for the authors to be the sole named individuals responsible for ensuring data access.

We will update your Data Availability statement to reflect the information you provide in your cover letter.

*As per our current data availability statement, we have provided readers with access to the raw sequencing files generated as part of this study through NCBI/SRA via BioProject PRJNA943266. At this time, further data cannot be shared due to privacy considerations, given the Biosecurity mandate of the involved organizations. However, we would be happy to provide more information on a case-by-case basis to interested parties who meet our security requirements. Interested groups can contact Cindi Corbett (*[*cindi.corbett@phac-aspc.gc.ca*](mailto:cindi.corbett@phac-aspc.gc.ca)*) for further information.*

4. PLOS requires an ORCID iD for the corresponding author in Editorial Manager on papers submitted after December 6th, 2016. Please ensure that you have an ORCID iD and that it is validated in Editorial Manager. To do this, go to ‘Update my Information’ (in the upper left-hand corner of the main menu), and click on the Fetch/Validate link next to the ORCID field. This will take you to the ORCID site and allow you to create a new iD or authenticate a pre-existing iD in Editorial Manager. Please see the following video for instructions on linking an ORCID iD to your Editorial Manager account: <https://www.youtube.com/watch?v=_xcclfuvtxQ>

*I have linked the appropriate ORCID ID as requested.*

Reviewer's Responses to Questions

**Comments to the Author**

Review Comments to the Author

Please use the space provided to explain your answers to the questions above. You may also include additional comments for the author, including concerns about dual publication, research ethics, or publication ethics. (Please upload your review as an attachment if it exceeds 20,000 characters)

Reviewer #1: -It is confusing without the line numbers and page numbers to follow and write the comments.
-What was the main conclusion? It needs to be clearly written in the abstract
-Abstract: Line 12: WIMP: acronyms should be written in full upon the first appearance.
-Abstract: Line 3: proofread the grammar: “MinIONs small footprint”.
-Abstract: Line 16: remove the comma.
-In the impact statement: this sentence is not understood and needs to be re-written: “showed less in the way of sequencing bias”. And the impact statement needs to be clear about the mai conclusions of the study.

*Thank you for these valuable notes. We have addressed those specifically listed here and have revised the rest of the manuscript for grammatical edits as per this recommendation.*

-The resolution of Figure 1 needs to be better to be read easily. Can the scientific names of the strains on the x axis be italicized? Also figure 2 labels need to be larger and with better resolution.

*Thanks to the reviewer for pointing this out. We have revised the images with greater resolution, and are anxious to hear whether we have addressed this concern sufficiently.*

-Grammatical proofreading is needed throughout the text.

*We have addressed the specific grammatical issues listed above and have revised the rest of the manuscript for grammatical edits as per this recommendation.*

Reviewer #2: The manuscript describes a procedure that will be the alternative or new technique for the pathogen or disease detection in samples. More study should be done for the validation of the methods but this paper it's a good start. I suggest adding a figure describing/outlining the workflow of the samples each group analysed.

*We have included a summary flowchart outlining our sample handling processes as part of Supplementary Figure 1.*
